# Antimicrobial activity of *Prunella Vulgaris* extracts against multi-drug resistant *Escherichia Coli* from patients of urinary tract infection

**DOI:** 10.12669/pjms.343.14982

**Published:** 2018

**Authors:** Sumra Komal, Syed Asif Jahanzeb Kazmi, Junaid Ali Khan, Mashkoor Mohsin Gilani

**Affiliations:** 1 Sumra Komal, Pharm-D, M. Phil (Pharmacology). Institute of Pharmacy, Physiology and Pharmacology, University of Agriculture, Faisalabad, Pakistan; 2 Syed Asif Jahanzeb Kazmi, MBBS, M.Phil, Ph.D Scholar (Pharmacology). Associate Professor, CMH Institute of Medical Sciences, Department of Pharmacology and Therapeutics, Bahawalpur, Pakistan; 3 Junaid Ali Khan, DVM, MSc, M.S., Ph. D (Endocrinology). Institute of Pharmacy, Physiology and Pharmacology, University of Agriculture, Faisalabad, Pakistan; 4 Mashkoor Mohsin Gilani, DVM, Ph.D (Molecular Microbiology). Assistant Professor, Institute of Microbiology, University of Agriculture Faisalabad, Pakistan

**Keywords:** Antibacterial activity, *Escherchia coli*, Muti-drug resistant, Minimum Inhibitory concentration, Prunella vulgaris, Urinary tract infection

## Abstract

**Background and Objective:**

*Escherichia Coli* is the most common etiological agent of UTI and accounts for more then 100, 0000 hospitalization annually. The objective of this study was to investigate the antimicrobial activity of aqueous and ethanolic extracts of *Prunella vulgaris* against *E. coli* from urinary tract infection patients.

**Methods:**

Urine samples of forty four suspected patients from Tertiary Care Hospital Faisalabad were used in this study. Ethanolic and aqueous extracts of *Prunella vulgris* (PV), a medicinal plant was evaluated for its ability to inhibit the growth of 38 resistant isolates of *Escherichia coli* strains and compared to Ciprofloxacin, Ofloxacin, Cefixime and Tobramycin by well diffusion method. Minimum inhibitory concentration was measured by using broth micro dilution method.

**Results:**

PV showed antibacterial activity against *Escherichia coli* strains, however Tobramycin at 10 microgram (10μg) inhibited the resistant *E. coli* to a greater extent as compared to other antibiotics and was resistant to twice less number of strains, about 82% of *E. coli* isolates have MDR pattern.

**Conclusion:**

Ciprofloxacin has more efficacy than PV and no synergistic effect with extracts of PV. Cefixime is least efficacious against resistant *E. coli*, however it has synergistic effect with extracts of PV.

## INTRODUCTION

Urinary tract infections (UTIs) are considered to be the second most frequent bacterial infection among human and is widely responsible for nosocomial infections in both developed and undeveloped countries.^1^ The studies showed that about 5% of men and 40–50% of women develop UTI in their lifetime and it accounts for more than 100,000 hospitalizations and spends about 1.6 billion dollars in medical expenses each year.^2^ UTI is caused by a variety of pathogens and uropathogenic *Escherichia coli* which accounts for approximately 75% of the isolates and being the most common etiological agent followed by *Klebsiella pneumonia*.^3^ However, emergence of resistance to commonly used antibiotics in UTI has become a great challenge.

Multidrug resistance is defined as an antimicrobial resistance shown by certain microorganisms to a number of antimicrobial drugs. Antimicrobial resistance is related to high rate of morbidity, mortality, increased treatment cost, hospital stay and decreased effectiveness of antimicrobial agents.^4^

Mostly herbal medicines are used in the development of new medicines and bring significant economic support in the treatment of many diseases.^5^ *Prunella vulgaris* (PV), a perennial plant widely distributed in Europe and East Asia, is commonly known as “self-heal” due to its quick wound healing effects.^6^ Keeping in view the properties of PV, the objective of present study are there for to investigate the antimicrobial activity of aqueous and ethanolic extracts of *Prunella vulgaris* against *E. coli* from urinary tract infection patients.

## METHODS

### Samples Collection

Urine samples of forty four (n=44) patients collected aseptically from Urology ward at Tertiary Care Hospital Faisalabad after informed written consent had been obtained. Urine samples were obtained in sterile container and carried under the refrigeratory condition and stored at 4°C until used further. Sample sizes were calculated by using sample size calculator. It was an open-labeled prospective cross-sectional study.

### Inclusion and Exclusion Criteria

Inclusion criteria include the bacterial strains that are resistant to more than one antibiotic and exclusion criteria for those strains which are sensitive to all antibiotics.

### Bacterial Culture and Identification

Urine samples were spread on MacConkey agar plates and overnight incubation of these plates at 37°C. Pink colonies on MacConkey agar plates identified the presence of *Escherichia coli*. Biochemical identification of *E. coli* was done by Rap ID one Panel (Remel, UK) according to manufacturer’s guidelines.

### Antimicrobial Sensitivity Testing

All clinical isolates were tested against Tobramycin (10μg), Ciprofloxacin (5μg), Cefixime (5μg) and Ofloxacin (5μg) using Kirby-Bauer disk diffusion method (CLSI, 2012). Isolates with resistant to more than two antibiotics were selected for further processing.

### Plant Material

Aerial parts of the *P. vulgaris* plant were obtained and identified by the Department of Botany, University of Agriculture Faisalabad, Pakistan.

### Plant Extract Preparation: Prunella vulgaris Aqueous Extract

Dried *Prunella vulgaris* aqueous extraction were prepared by grinding it followed by soaking in water (1 L) for 2 hour and then boiled in distilled water for 2 hr at 100°C. The extract was cooled at room temperature and supernatant was then filtered through Whatman No.1 filter paper. Residues were reconstituted with distilled water and were used in the study followed by freeze drying.^7^

### Prunella vulgaris Ethanolic Extract

Plant dried material was grinded and extracted with 95% ethanol by using Soxhlet extractors, the extract was concentrated by rotary evaporation at less than 30°C and was lyophilized.^8^

### Minimum Inhibitory Concentration of Plant Extracts

The minimum inhibitory concentration (MIC) of Quinolones, Cephalosporins and plant extracts were analyzed by using broth microdilution method.^9^ According to the Clinical Laboratory Standards Institute, guidelines 2011, serial dilutions of plant materials with significant antimicrobial activity with 5 × 105 (CFU) /mL were prepared.^10^ Ethanolic extract were diluted two folds to develop a series of concentrations by using brain heart infusion (BHI) broth.MIC was evaluated at lowest conc. of PV extract i.e. 0.125 μg/μL without any visible bacterial growth in wells of 96 well plates.

### Statistical Analysis

Statistical analysis was done by using SPSS 16.0, t-test and one way ANOVA was used to determine the significance difference among all the experimental groups (p≤0.05).

## RESULTS

Out of 44 Samples, 38 (86.4%) urine samples carried *E. coli* with high rates of MDR *E. coli* (82%), (Table-I). The zone of inhibition against resistant strains of *E. coli* was measured, tobramycin at concentration 10(μg) had a zone of inhibition 16.16(±0.822) mm as compared to ciprofloxacin, ofloxacin, cefixime with zone of inhibition at concentration of 5 μg, 10.79 (±1.329), 10.02(±1.107) and 7.37(±0.54) respectively. Therefore tobramycin at 10 microgram inhibited the resistant *E. coli* to a greater extent as compared to other antibiotics (Table-II).

**Table-I:** Percentage multi-drug resistance of isolated samples.

Total urine samples	*E. coli* positive samples	*E. coli* negative samples	MDR *E. coli* strains
Percentage	86.4%	13.6%	82%

**Table-II:** Antibiotics zones of inhibition against *Escherichia coli* strains.

	Number of Observation	Mean Zone of Inhibition (mm)	Std. Deviation	Std. Error Mean
Tobramycine 10 Microgram	3 7	16.16	4.997	± 0.822
Cefixime 5 Microgram	3 8	7.37	3.329	±0.540
Ofloxacin 5 microgram	3 8	10.02	6.823	±1.107
Ciprofloxacine 5 Microgram	3 8	10.79	8.194	±0.1329

The mean MIC of ciprofloxacin, ciprofloxacin plus aqueous extract of *Prunella vulgaris*, ciprofloxacin with ethanolic extract of *Prunella vulgris* and Ciprofloxacin with both aqueous and ethanolic extract of *Prunella vulgris* is 0.5 ± 0.00 µgm/µl, 2 ± 0.00µgm/µl, 1.33 ± 0.33µgm/µl, 1.83 ± 1.09 µgm/µl, 2.16 ± 1.0179µgm/µl and 1.33 ± 0.33µgm/µl respectively. Therefore Ciproflaxacin alone has greater zone of inhibition on lesser dose 0.5 μg/μL as compared to *Prunella vulgaris* extracts (both aqueous and ethanolic) and in combination with Ciprofloxacin. Therefore it is suggested that *Prunella vulgaris* has no synergistic effect with Ciproflxacin. (Table-III).

**Table-III:** Cumulative data of MIC of Ciprofloxacine and *P. vul* extracts against ATCC 25922, UR- 1 and UR- 110 (resistant strains of *E. coli*).

	Concentration	Ciprofloxacine	Aqueous Extract Prunella vulgaris	Ethanolic Extract Prunella vulgaris	Ciprofloxacine Aqueous Extract Prunella vulgaris	Ciprofloxacin Ethanolic Extract Prunella vulgaris	Ciprofloxacin Aqueous Extract + Ethanolic Extract Prunella vulgaris
Valid	3	3	3	3	3	3	3
Missing	0	0	0	0	0	0	0
Mean		0.5000	2.0000	1.3333	1.8333	2.1667	1.3333
Std. Error of Mean		0	0	0.3333	1.09291	1.01379	0.3333
Median		0.5000	2.0000	1.0000	1.0000	2.0000	1.000
Mode		0.5	2.00	1.00	0.5	0.5	1.00
Std. Deviation		0.00	0.000	0.57735	1.89297	1.75594	0.57735

The mean MIC of Cefixime, aqueous extract of *Prunella vulgris*, ethanolic extract of *Prunella vulgaris*, Cefixime plus aqueous extract of *Prunella vulgaris*, Cefixime with ethanolic extract of *Prunella vulgaris* and Cefixime with both aqueous and ethanolic extract of *Prunella vulgris*8.83 ± 0.00 µgm/µl, 2 ± 0.00 µgm/µl, 2 ± 0.00 µgm/µl, 1.33 ± 0.33 µgm/µl, 1.5 ± 0.50µgm/µl and 4.0 ± 0.00 µgm/µl respectively. Therefore Cefiximewith aqueous extract of *Prunella vulgaris* has greater zone of inhibition on lesser dose as compared to *Prunella vulgaris* extracts (both aqueous and ethanolic) andCefixime alone. Therefore it is suggested that Cefixime plus aqueous extract of *Prunella vulgaris* has synergistic effect with cefixime (see Table-IV).

**Table-IV:** Cumulative data of MIC of Cefixime and *P. vul* extracts against ATCC 25922, UR-1 and UR- 110 (resistant strains of *E. coli*)

	Concentration	Cefixime	Aqueous Extract Prunella vulgaris	Ethanolic Extract Prunella vulgaris	Cefixime Aqueous Extract Prunella vulgaris	Cefixime Ethanolic Extract Prunella vulgaris	Cefixime Aqueous Extract Ethanolic Extract Prunella vulgaris
Valid	3	3	3	3	3	3	3
Missing	0	0	0	0	0	0	0
Mean		8.8333	2.0000	2.0000	1.3333	1.5000	4.0000
Std. Error of Mean		8.08462	0.00000	0.00000	0.33333	0.50000	0.00000
Median		1.0000	2.0000	2.0000	1.0000	2.0000	4.0000
Mode		0.50^a^	2.00	2.00	1.00	2.00	4.00
Std. Deviation		1.40030E1	0.00000	0.00000	0.57735	0.86603	0.00000
Variance		196.083	0.000	0.000	0.333	0.750	0.000

## DISCUSSION

Antibiotics resistance to urinary tract pathogens has been raising globally from many years.^11^ Multi-drug resistant bacteria are serious threat in clinical health settings and very challenging to treat infectious disease.^12^ From current study it was found that 82% of clinical isolates of *Escherichia coli* have multi drug resistance pattern. Increased rate of prevalence in the present study indicate the irrational use of antibiotics in clinical practice. Approximately 80% of antimicrobial administration is unnecessary. This misuse of antimicrobials plays a significant role in the development of resistance among microorganisms and produce considerable adverse effects on human health.^13^ High prevalence rates of antibiotic-resistant strains of *Escherichia coli* complicate the management of urinary tract infections.^14^ In the present study antibiotic sensitivity testing was done by using four antibiotics include Ciprofloxacin, Ofloxacin, Cefixime and Tobramycin and found that Tobramycin (10μg) have higher zone of inhibition as compared to Fluoroquinolones and Cephalosporins which are commonly used for the treatment of urinary tract infection or a number of other bacterial infections.^15^ From previous studies it was found that Fluoroquinolones have lost their activity against MDR gram negative bacteria and it is very difficult to restore it, however, the use of tobramycin as an adjuvant can help to improve Fluoroquinolones activity.16 The irrational use of these medicines is the leading cause of development of resistance.

Resistance to antimicrobial is more threatening because only a limited number of new antimicrobial agents have been developed.^17^ Natural herbs have been used in treating a variety of illness and play a major role in the preparation of modern medicine. 18,19 In the current study an attempt is made to find out the activity of *Prunella vulgaris* against MDR strains of *E. coli*. From previous studies it has been revealed that PV can inhibit the bacterial growth. In this study ethanolic and aqueous extract of *P. vulgaris* was prepared the average MIC of aqueous extract was 2μg/μl which is more than the MIC of ethanolic extract of *P. vulgaris* that was 1 μg/μl. Antibacterial combination most commonly used by the practitioners to treat bacterial infection as the synergistic effect of two or more medicine improve the overall effect of conventional antibiotics by increasing its efficacy, reducing toxicity and provide broad spectrum activity against microbes.^19^ Resistance behaviors of MDR *E. coli* were modified by using *P. vulgaris* plant extract in combination with conventional medicine. From the current study, it was found that *P. vulgaris* along with conventional antibiotics against UTI have shown better results by exhibiting average MIC of 0.5μg/μl.

## CONCLUSION

Aqueous and ethanolic extract alone and in combination have positive effect against multi-drug resistant *E. coli* strain isolated from patients with urinary tract infection. In this study it is suggested that;

1. *E. coli* is positive to 86.4% of urine samples. Focus on MDR *E. coli*
2. Tobramycin has greater efficacy against resistant *E. coli* as compared to other antibiotics.
3. Ciprofloxacin has more efficacy than aqueous or ethanolic extract of PV.
4. Ciprofloxacin has no synergistic effect with PV extracts.
5. Cefixime is least efficacious against resistant *E. coli* however it has synergistic effect with extracts of PV.

Hence, it is concluded that *P vulgaris* can be used as supportive therapy along with the standard antibiotics used to treat UTI.

### Author`s Contribution

**JAK:** Supervised the research work and study planning.

**SAJK:** Statistical analysis, manuscript writing & editing of manuscript.

**SK, JAK, MMG:** Conceived & design, data collection, data entry and manuscript writing.

**SK, SAJK:** Review and final approval of manuscript.
